# Supplementary material for: Multi‐dataset identification of innovative feature genes and molecular mechanisms in keratoconus
Source: J Cell Mol Med. 2024 Sep 19;28(18):e70079. doi: 10.1111/jcmm.70079 (PMC11412914; doi:10.1111/jcmm.70079)
Supplement: Supplementary file 2 — Material S2. [file JCMM-28-e70079-s002.zip › 7.GSEA analysis/4.MYRF/KEGG_ALLOGRAFT_REJECTION.html]

Details for gene set KEGG\_ALLOGRAFT\_REJECTION[GSEA]

|  || Dataset | MYRF.group.cls#h\_versus\_l |
| Phenotype | group.cls#h\_versus\_l |
| Upregulated in class | l |
| GeneSet | KEGG\_ALLOGRAFT\_REJECTION |
| Enrichment Score (ES) | -0.5773148 |
| Normalized Enrichment Score (NES) | -1.4036775 |
| Nominal p-value | 0.077380955 |
| FDR q-value | 0.24256371 |
| FWER p-Value | 0.879 |
Table: GSEA Results Summary

  

Fig 1: Enrichment plot: KEGG\_ALLOGRAFT\_REJECTION      
 Profile of the Running ES Score & Positions of GeneSet Members on the Rank Ordered List

  

| SYMBOL | TITLE | RANK IN GENE LIST | RANK METRIC SCORE | RUNNING ES | CORE ENRICHMENT || 1 | CD86 | na | 355 | 0.626 | 0.0634 | No |
| 2 | IL12A | na | 1851 | 0.369 | 0.0637 | No |
| 3 | HLA-DOA | na | 8167 | 0.134 | -0.1025 | No |
| 4 | CD28 | na | 12283 | 0.059 | -0.2142 | No |
| 5 | HLA-B | na | 12962 | 0.051 | -0.2278 | No |
| 6 | HLA-F | na | 16261 | 0.015 | -0.3211 | No |
| 7 | HLA-E | na | 16263 | 0.015 | -0.3193 | No |
| 8 | FASLG | na | 16299 | 0.015 | -0.3186 | No |
| 9 | HLA-C | na | 17654 | 0.003 | -0.3573 | No |
| 10 | HLA-A | na | 20084 | -0.023 | -0.4246 | No |
| 11 | HLA-DRB5 | na | 20183 | -0.024 | -0.4246 | No |
| 12 | HLA-G | na | 20210 | -0.024 | -0.4225 | No |
| 13 | IL12B | na | 22624 | -0.054 | -0.4857 | No |
| 14 | GZMB | na | 22901 | -0.058 | -0.4869 | No |
| 15 | HLA-DRB3 | na | 23156 | -0.062 | -0.4869 | No |
| 16 | HLA-DQB1 | na | 24038 | -0.076 | -0.5033 | No |
| 17 | HLA-DRB1 | na | 24046 | -0.077 | -0.4945 | No |
| 18 | HLA-DOB | na | 24105 | -0.078 | -0.4870 | No |
| 19 | IL5 | na | 26211 | -0.117 | -0.5339 | No |
| 20 | CD80 | na | 26452 | -0.122 | -0.5264 | No |
| 21 | HLA-DMA | na | 27498 | -0.148 | -0.5392 | No |
| 22 | FAS | na | 28092 | -0.163 | -0.5371 | No |
| 23 | IL10 | na | 28824 | -0.186 | -0.5363 | No |
| 24 | HLA-DRB4 | na | 30247 | -0.232 | -0.5500 | Yes |
| 25 | CD40LG | na | 30708 | -0.251 | -0.5338 | Yes |
| 26 | PRF1 | na | 31281 | -0.279 | -0.5175 | Yes |
| 27 | HLA-DPA1 | na | 32053 | -0.320 | -0.5021 | Yes |
| 28 | CD40 | na | 32572 | -0.356 | -0.4752 | Yes |
| 29 | HLA-DRA | na | 33031 | -0.390 | -0.4425 | Yes |
| 30 | IL4 | na | 33045 | -0.391 | -0.3969 | Yes |
| 31 | IFNG | na | 33150 | -0.402 | -0.3527 | Yes |
| 32 | HLA-DMB | na | 33256 | -0.414 | -0.3070 | Yes |
| 33 | IL2 | na | 33402 | -0.431 | -0.2605 | Yes |
| 34 | HLA-DQA2 | na | 34217 | -0.576 | -0.2162 | Yes |
| 35 | TNF | na | 34328 | -0.609 | -0.1478 | Yes |
| 36 | HLA-DPB1 | na | 34331 | -0.609 | -0.0762 | Yes |
| 37 | HLA-DQA1 | na | 34602 | -0.745 | 0.0036 | Yes |
Table: GSEA details [plain text format]

  

Fig 2: KEGG\_ALLOGRAFT\_REJECTION      
 Blue-Pink O' Gram in the Space of the Analyzed GeneSet

  

Fig 3: KEGG\_ALLOGRAFT\_REJECTION: Random ES distribution      
 Gene set null distribution of ES for **KEGG\_ALLOGRAFT\_REJECTION**
